# The impact of socioeconomic status, general health and oral health on Health-Related Quality of Life, Oral Health-Related Quality of Life and mental health among Polish older adults

**DOI:** 10.1186/s12877-021-02716-7

**Published:** 2022-01-03

**Authors:** Barbara Malicka, Katarzyna Skośkiewicz-Malinowska, Urszula Kaczmarek

**Affiliations:** Department of Conservative Dentistry With Endodontics, Wroclaw Medical University, Wrocław, Poland

**Keywords:** Elderly, Quality of Life, EQ-5D, PHQ-9, OHIP-14, Oral Health Impact Profile 14

## Abstract

**Background:**

The study aims to evaluate the impact of socioeconomic status, general health and oral health parameters on Health-Related Quality of Life (HRQoL), Oral Health-Related Quality of Life (OHRQoL) and mental health in elderly urban residents of South-Western Poland.

**Methods:**

The 500 residents of Wroclaw, aged 65 and older provided demographic and personal information as well as their medical history. A patient's oral condition were determined based on the clinical oral examination.Quality of Life was assessed using Euro-Quality of Life (EQ-5D), Oral Health Impact Profile-14 (OHIP-14) and Patient Health Questionnaire (PHQ-9).The association between exposure (socioeconomic status, general health and oral health) and outcome (HRQoL, OHRQoL and mental health variables) were analyzed with the use of four models: P – Poisson model, NB-Negative Binomial model, ZIP – Zero Inflated Poisson model, ZINB – Zero Inflated Negative Binomial model.

**Results:**

The best model turned out to be the ZINB model, in which a negative binomial distribution in the count equation is assumed. In this model, only 13 independent variables had a significant effect on HRQoL, OHRQoL, and mental health. HRQoL assessed with the EQ-5D is significantly influenced by: living conditions 0.133 (95% CI: 0.001, 0.267, *p* = 0.049), income -0.348 (95%CI: -0.466, -0.230, *p* < 0.001), diabetes mellitus 0.437 (95%CI: 0.250, 0.624, *p* < 0.001), myocardial infarction 0.454 (95% CI: 0.151, 0.757, *p* = 0.003), stroke 0.543 (95%CI: 0.094, 0.992, *p* = 0.018) and renal disease 0.466 (95% CI: 0.206, 0.726, *p* < 0.001). Factors negatively affecting OHRQOL are: the need for oral treatment 0.278 (95%CI: 0.104, 0.452, *p* = 0.002), the number of missing teeth 0.053 (95%CI: 0.039, 0.067, *p* < 0.001) and gender 0.271 (95%CI: 0.015, 0.527, *p* = 0.038) and age -0.025 (95%CI: -0.042, -0.008, *p* = 0.003). An important factor influencing the level of depression assessed by the PHQ-9 questionnaire may be the material condition -0.225 (95%CI: -0.349, -0.101, *p* < 0.001). It should be emphasized that living with other people may be a factor that significantly increases the probability of avoiding the occurrence of depression symptoms.

**Conclusion:**

The study concerning elderly residents of the macroregion in Poland found the impact of socioeconomic, general health and oral health parameters on Health-Related Quality of Life, Oral Health-Related Quality of Life and mental health. Research on the quality of life of the elderly at the local level allowed to assess the factors linked to quality of life of older adults.

## Background

Population ageing is undeniable, especially in highly developed countries. In Poland, the share of the elderly population, i.e. people aged 65 and more, exceeds 10% in the general population structure. Therefore, Polish society is considered a society of "advanced old age" [1–3]. The longer life expectancy of people and the decline in birth rates are the main reasons for an increased proportion of the elderly in society. The singularization in old age is a feature of demographic ageing and it is reflected by the high proportion of the elderly who reside in one-person households. Another feature of population ageing in Poland is the feminization of old age, expressed in the predominance of elderly women. The so-called “double ageing”, which means a faster rate of increase in the proportion of the population aged 80 and more [4], is also observed. According to projections, the proportion of the population aged 65 and more will increase steadily. In 2035, it is expected to reach 23.2%, with a slightly higher level in urban areas (24.3%) than in rural areas (21.7%). In 2035, the highest percentage of people aged 65 and more will be reported in the following territorial units for statistics (NUTS 1): Eastern Region – PL8 (24.3%) and South-Western Region – PL5 (24.2%). The highest proportion of people aged 80 and more will be reported in the South-Western Region – PL5 (7.6%) and Central Region – PL7 (7.4% [1].

Cities are projected to have a much higher proportion of people aged 80 and more, which also indicates a potentially greater need for. social support and healthcare provision. Modern cities should cater for the needs of all social groups, including the elderly, to ensure an improved quality of life for their residents. There is a need for observational and interventional studies on the quality of life of the elderly at the local level to enable formulating conclusions and recommendations applicable at the local level and define public health interventions to improve the quality of life of the post-working age group [1].

Various local, national and international organisations have long been strongly involved in the development of strategies aimed at improving the quality of life of the elderly. The definition developed by the World Health Organization Quality of Life (WHOQOL Group) is among those presenting a holistic approach to the quality of life. According to this definition, quality of life is an individual’s perception of their position in life in the context of their culture and value systems in relation to their goals, expectations, standards and concerns. It is a broad concept that is comprehensively influenced by an individual's physical health, mental state, level of independence, social relationships, personal beliefs and attitude towards the surrounding environment. When assessing the quality of life of the elderly, however, the concept of health-related quality of life (HRQoL) should be examined more broadly, and the impact of oral health on quality of life (oral health-related quality of life – OHRQoL) should not be underestimated either [5, 6]. It is important to recognize the relationship between oral health and quality of life because interactions between these two elements impact the daily life of an individual on functional, social and psychological levels. Studies evaluating OHRQoL allow a better understanding of the interactions between the perception of oral health, external environment, individual characteristics, health-related behaviors as well as objective and subjective health [7, 8] The approach offered takes into account the impact of factors such as illness, disability and natural age-related limitations on the perception of the quality of life. The prevalence of mental disorders is on the rise among the elderly and a strong link has been found between mental and physical health even after controlling for confounders. Understanding the potential pathways through which mental health affects physical health and vice versa could have important implications for the design of health policies [9]. In this perspective, HRQoL is considered a state of well-being that is influenced by the ability to cope with daily tasks, physical, mental and social well-being, satisfaction with functioning in all areas of life, as well as maintaining control over illness.

Given the above-mentioned demographic projections and the fact that the process of demographic ageing of the population will be uneven, i.e. it will shape the demographic structure of urban residents to a greater extent, a study concerning the elderly population in Poland was conducted in a macro-region PL5 (NUTS 1) in the city of Wroclaw – PL514 (NUTS 3). The study aimed to evaluate the impact of socioeconomic status (SES), general and oral health parameters on HRQoL, OHRQoL and mental health in elderly urban residents of South-Western Poland.

## Materials and Methods

### The study protocol

The presented observational study was conducted as part of the project "Oral health and quality of life within old ages – a cross-sectioned study in Germany and Poland" in cooperation between Municipal Council and the University Hospital Carl Gustav Carus in Dresden, Germany. The study was supported with funds from the Municipal Council as part of the project “Oral Health and the Quality of Life of Elderly Residents of Wroclaw” (financial agreement P/ZJU/1/2015–2017). The STROBE (Strengthening the Reporting of Observational Studies in Epidemiology) guidelines were followed [10]. The study protocol was approved by the Bioethics Committee of the Wroclaw Medical University (permission no. KB 420/2015) in accordance with the Declaration of Helsinki. Participation in the study was voluntary and anonymous. All collected data were treated confidentially.

The data concerning the total number of residents aged 65 and more were obtained from Statistics Poland [11]. The sample size was calculated based on the data concerning the number of individuals in this age group who live in the city, accounting for 0.0035% of the population of Wroclaw. In 2015, the overall population of Wroclaw was 635,759 (339,105 women and 296,654 men), while the population of 65-year-olds and older people was 144,317. With such an assumption, a 95% level of confidence and a ± 5% margin of error were set, and a minimum sample size consisted of 384 participants [12].

The inclusion criteria were as follows: age ≥ 65, place of residence (local resident, Wroclaw, Poland), ability to communicate, written consent to participate in the study. The exclusion criteria included concurrent systemic diseases in which periodontal probing leading to transient bacteraemia might have posed a risk to the patient's overall health; failure to give written consent to participate in the survey; the occurrence of mental disorders which render the completion of the questionnaire impossible. All participants gave written informed consent and completed the questionnaire. The participants who did not meet the inclusion criteria were excluded from the study.

The study involved randomly selected participants aged 65–99 (n = 1338), both men and women. However, 41.0% (n = 549) of them did not respond to the study invitation, 6.6% (n = 88) did not give written consent to participate in the study and 15.0% (n = 201) were excluded due to the presence of systemic concurrent diseases in which periodontal probing, leading to transient bacteraemia, might have posed a risk to the patient's overall health. The above-mentioned risk is particularly related to the participants diagnosed with cardiovascular diseases (patients with artificial heart valves, heart transplant, congenital heart diseases or infective endocarditis), blood diseases (thrombocytopenia, haemophilia, von Willebrand disease), viral diseases (hepatitis B and C, AIDS), as well as participants with multi-drug resistant organisms (MDRO). A total of 500 participants were included in the study.

### Sociodemographic and socioeconomic status (SES).

The participants provided their demographic and personal information. The collected data included date of birth, gender, marital status, level of education, monthly income (based on the national average income per family member in the year of study).

### General health status

Information concerning self-reported systemic diseases was collected with the use of a semi-structured questionnaire.

### Oral health parameters

The evaluation of oral health was conducted by two examiners (with the inter-examiner kappa coefficient of 0.874 and the intra-examiner kappa coefficient of 0.870). Coronal caries and root caries were evaluated according to the WHO criteria, DMFT (decayed, missing and filled teeth) values and their components. The periodontal condition was assessed in individuals who had more than 2 natural teeth by measuring bleeding on probing (BoP) in 6 sites, gingival pocket depth (PD), clinical attachment loss (CAL) and tooth mobility according to the Miller index [13]. A patient's treatment needs were determined based on the clinical oral examination and they were categorised on a 5-grade scale: no treatment need, preventive treatment, prompt treatment, immediate treatment, or referral.

Dental prosthetic status was assessed by taking into consideration both the number of functional tooth units (FTUs) of natural and artificial teeth on implant-supported fixed dentures (non-FTUs) and the information on whether the participants wore partial or complete removable dentures. Furthermore, oral dryness was examined according to the Challacombe scale (Clinical Oral Dryness Score – CODS). The level of oral dryness was categorised based on the number of symptoms observed as mild (1–3), moderate (4–6) or severe [14, 15].

### Psychometric parameters

The Euro-Quality of Life Questionnaire (EQ-5D) is a generic questionnaire created in 1987 by the EuroQoL Group. It consists of two parts: a descriptive part assessing HRQoL in five categories and a visual analogue scale (EQ-VAS). Each patient rated their level of mobility, self-care, ability to perform daily activities, pain and discomfort experienced, anxiety and depression on a three-point scale: no problems (1 point), minor problems (2 points), major problems (3 points). Then, the patients rated their current health status on a 0–100 VAS (0 – the worst imaginable health; 100—the best imaginable health) [16, 17].

The impact of OHRQoL was measured using the previously validated Polish version of the Oral Health Impact Profile-14 (OHIP-14) [18]. The questionnaire consisted of 14 questions related to experienced problems: trouble with pronouncing words, worsened taste, pain, discomfort while eating, self-consciousness, emotional tension, unsatisfactory diet, interrupted meals, difficulty with relaxing, embarrassments, irritability, inability to complete everyday tasks, reduced satisfaction with life, complete inability to function. The questions corresponded to 7 dimensions: functional limitation, pain, psychological discomfort, physical disability, psychological disability, social disability and handicap. The frequency of occurrence was assessed using the five-point Likert scale: 0 – never, 1 – rarely, 2 – occasionally, 3 – frequently, 4 – very often. All values were summed up to calculate the total OHIP-14 score that could vary between 0 and 56; the higher the OHIP-14, the poorer the OHRQoL [18].

Depression was determined using the Patient Health Questionnaire-9 (PHQ-9). It is a tool that can be used by doctors of specialities other than psychiatry for screening patients for depression. The questionnaire was filled out by the patients after they had been given instructions. The answers to the questions concerning patients’ well-being in the past 2 weeks were categorised according to the frequency of occurrence on a scale from 0 to 3 (0 — not at all, 1 — several days, 2 — more than half the days, 3 — nearly every day). The number of scores determined the severity of depression: 0–4 – no depressive symptoms or minimal symptoms, 5–9 – mild symptoms, 10–14 – moderate symptoms, 15–19 – moderately severe symptoms, 20–27 – severe symptoms [19, 20].

### Statistical analysis

The results obtained from both questionnaires and clinical examinations were statistically analysed using STATISTICA v. 13 (TIBCO, Software Inc., USA). Continuous variables were reported as mean values ± standard deviation or, if not normally distributed, as median and inter-quartile range. Categorical variables were reported as numbers and percentages. The normality of empirical distributions of the quantitative variables was found using the Kolmogorov–Smirnov test.

An association between dependent variables (3) and independent variables (41) was analyzed with the use of four models (P – Poisson model, NB-Negative Binomial model, ZIP – Zero Inflated Poisson model, ZINB-Zero Inflated Negative Binomial model). Due to the large dispersion of the individual EQ-5D, OHIP-14 and PHQ-9 results and the fact that the variances were higher than the mean values, the Poisson distribution and the negative binomial distribution were used for the analysis of variables. Parameters of mathematical models for the number of patients: Poisson (P), negative binomial (NB), Zero Inflated Poisson (ZIP) and Zero Inflated Negative Binomial (ZINB) were adjusted to the observed values using the "pscl" package of R for Windows (Version 4.0.4). Since the logistic regression coefficients only show the direction of the influence of the independent variables on the dependent variable, and not its strength, the values of the odds ratios for each variable were estimated. It was presented how many percentage points the dependent variable will change in the case of a unit increase in the independent variable. Because the distribution histograms of EQ-5D, OHIP-14 and PHQ-9 codes were characterized by strong asymmetry (positive skew) and the variances of count data were larger than the mean values, variables were described with Poisson distribution and negative binomial distribution. Additionally, a very high number of 0-codes was observed, which is why the following count models with a large number of zero values were used for modelling the count of patients: ZIP (*Zero Inflated Poisson model*) and ZIBB (*Zero Inflated Negative Binomial model*). In all statistical tests, the critical value was set at *p* < 0.05.

## Results

### Sociodemographic characteristics

A total of 500 individuals participated in the study; 64.0% of them were women and 36.0% were men. The median age in the study group was 73 (Q1 = 68, Q3 = 79). The majority of participants reported to have secondary (51.0%) or higher level of education (32.8%). They belonged to the middle or high economic class (45.2% and 27.4%, respectively). Most of them lived with their family members (62.0%) and stayed at home without any help (90.8%) (Table 1).

**Table 1:** Sociodemographic and socioeconomic status

Independent Variable	N(%)	Me (IQR) Min–Max
Gender:		
Women	64.0% (320)	
Men	36.0% (180)	
Age, years		
Me (IQR)		73 (68–79)
Min–Max		65–99
Living with:		
Alone	38.0% (190)	
Other people	62.0% (310)	
Living conditions		
Home without help	90.8% (454)	
Home with help	8.6% (43)	
Residence	0.6% (3)	
Number of persons in a household		
Me (IQR)		2 (1–2)
Min–Max		1–5
Income		
Low	25.6% (128)	
Medium	45.2% (226)	
High	27.4% (137)	
No answer	1.8% (9)	
Education level		
Primary	16.2% (81)	
Medium	51.0% (255)	
High	32.8% (164)	

### General health

In the study group, 62% of patients suffered from hypertension, 22% from diabetes and 18.6% from hypercholesterolaemia. Most participants (35.4%) suffered from at least three concurrent systemic diseases (Table 2).

**Table 2:** General health

Independent variables:	N (%)
Hypertension	304 (62.0%)
Hypercholesterolaemia	91 (18.6%)
Diabetes	108 (22.0%)
Infarction	25 (5.1%)
Heart failure	35 (7.1%)
Atrial fibrillation	18 (3.7%)
Stroke	10 (2.0%)
Asthma	32 (6.5%)
Reflux	48 (9.8%)
Ulcerative disease	9 (1.8%)
Kidney diseases	38 (7.8%)
Hyperthyroidism	21 (4.3%)
Hypothyroidism	55 (11.2%)
Cancer	27 (5.5%)
Osteoartritis	14 (2.9%)
Osteoporosis	13 (2.7%)
Rheumatoid arthritis	71 (14.5%)
Number of diseases	
none	14% (70)
1	23.6% (118)
2	27.0% (135)
3	35.4% (177)

### Oral health parameters

In the clinical examination, the average number of teeth in the mouth was 12.97 ± 9.5. The average number of teeth in the occlusion was 4.7 ± 4.8. A partial removable maxillary denture was worn by 30.8% of participants, while 20.0% of participants had a partial removable mandibular denture. A complete removable denture was worn by 25.0% and 22.6% of participants, respectively. Edentulism was found in 106 participants (21%). Oral dryness was observed in 32.8% of participants, out of which 29.6% suffered from mild oral dryness, 3.0% had moderate oral dryness and 0.2% suffered from severe oral dryness. Based on the clinical examination, more than 60% of the patients required professional preventive care. Approximately 30% of patients required dental treatment, most commonly due to caries (Table 3).

**Table 3:** Oral health status

**Oral health parameters**	Mean ± SD	% (n)		Me (Q1 – Q3)	Min – Max
Intervention urgency:	1.3 ± 0.7			1 (1—2)	0—4
no treatment need % (n)		10.4% (52)			
preventive treatment % (n)		54.8% (274)			
prompt treatment % (n)		30.2% (151)			
immediate treatment % (n)		4.4% (22)			
referral % (n)		0.2% (1)			
Oral dryness:	0.8 ± 1.2			0 (0—2)	0—5
no dryness		67.2% (336)			
a mild dryness		29.6% (148)			
moderate dryness		3.2% (16)			
Number of teeth in the mouth	12.97 ± 9.5		14 (10—28)		0—32
Number of missing teeth	19.0 ± 9.6		18 (10—28)		0—32
Number of teeth in the occlusion	4.7 ± 4.8		4 (0—9)		0—14
Number of unrepaired missing teeth	4.8 ± 7.0		2 (0—7)		0—32
Percentage of patients with dentures, % (n)		62.0% (310)			

### Psychometric examination

EQ-5D-3L Index was 0.849 ± 0.009 while the mean value of EQ-VAS was 66.7 ± 0.9 (Table 3). The results of the reliability analysis indicate good psychometric properties (Cronbach's alpha = 0.820).

The total OHIP-14 score was 8.01 ± 13.6 (Table 4). The results of the analysis concerning the OHIP-14 items in the study group of 500 elderly citizens indicate very good reliability (Cronbach's alpha = 0.964). In terms of domains, the participants most frequently reported physical pain (1.7 ± 2.5) and psychological discomfort (1.5 ± 2.5).

**Table 4:** Psychometric parameters-dependent variables

Questionnaire	Mean ± SD	Me (Q1 – Q3)	Min – Max
OHIP-14 functional limitation	1.0 ± 2.1	0 (0—1)	0—8
OHIP-14 physical pain	1.7 ± 2.5	0 (0—3)	0—8
OHIP-14 psychological discomfort	1.5 ± 2.5	0 (0—2)	0—8
OHIP-14 physicaldisability	1.2 ± 2.4	0 (0—1)	0—8
OHIP-14 psychological disability	1.1 ± 2.2	0 (0—1)	0—8
OHIP-14 social disability	0.7 ± 1.9	0 (0—0)	0—8
OHIP-14 handicap	0.8 ± 1.9	0 (0—0)	0—8
OHIP-14 (total scores)	8.0 ± 13.6	1 (0—9)	0—56
EQ-5D Mobility	0.164 ± 0.002	0.20 (0.12–0. 20)	0.01–0.26
EQ-5D Self-Care	0.182 ± 0.002	0.20 (0.20–0.21)	0.01–0.26
EQ-5D Usual Activities	0.181 ± 0.002	0.20 (0.20–0.21)	0.02–0.23
EQ-5D Pain / Discomfort	0.153 ± 0.002	0.14 (0.11–0.20)	0.02–0.26
EQ-5D Anxiety / Depression	0.169 ± 0.002	0.20 (0.12–0.20)	0.09–0.25
EQ-5D-3L Index	0.849 ± 0.009	0.89 (0.82—1)	0.09– 1
EQ-5D VAS	66.7 ± 0.9	70 (50—80)	5 – 100
Patient Health Questionnaire (PHQ-9):			
1.Little interest or pleasure in doing things?	0.3 ± 0.6	0 (0—0)	0—3
2.Feeling down, depressed, or hopeless?	0.6 ± 0.8	0 (0—1)	0—3
3.Trouble falling or staying asleep, or sleeping too much?	0.8 ± 1.0	1 (0—1)	0—3
4.Feeling tired or having little energy?	0.6 ± 0.9	0 (0—1)	0—3
5.Poor appetite or overeating?	0.4 ± 0.7	0 (0—1)	0—3
6.Feeling bad about yourself—or that you are a failure or have let yourself or your family down?	0.4 ± 0.6	0 (0—1)	0—3
7.Trouble concentrating on things, such as reading the newspaper or watching television?	0.3 ± 0.6	0 (0—0)	0—3
8.Moving or speaking so slowly that other people could have noticed?	0.2 ± 0.6	0 (0—0)	0—3
9.Thoughts that you would be better off dead, or of hurting yourself in some way?	0.0 ± 0.1	0 (0—0)	0—2
PHQ-9 (total scores)	3.6 ± 4.1	2 (0—6)	0—21

The mean value of PHQ-9 was 3.6 ± 4.1, which enabled the assignment of those participants to the minimally depressed group (Table 4). The patients most frequently complained of sleep problems (0.84 ± 1.01). The analysis of the distribution of participants in terms of the severity of depressive symptoms revealed that 69.2% of them had no depressive symptoms while mild, moderate and severe depressive symptoms were observed in 22.2%, 6% and 2.6% of participants, respectively.

In the Poisson regression model (Table 5), the following unit changes in the analysed parameters negatively affected the expected change of quality of life assessed with the EQ-5D questionnaire: female gender (25.6%), age (3.7%), living alone (16.2%), concomitant diabetes (36.0%), prior myocardial infarction (42.7%), heart failure (35.1%), prior stroke (61.2%), kidney diseases (140.7%), hypothyroidism (52.4%), as well as xerostomia (6.0%) and the number of missing teeth (3.2%). Income had a positive impact on the quality of life (-31.7%).

**Table 5:** Parameters of regression models and the impact of unit changes in individual variables on the expected number of patients (%) for the compared EQ-5D models

Independent variables	P	NB	ZIP		ZINB	
			**e1: Y > 0**	**e2: Y = 0**	**e1: Y > 0**	**e2: Y = 0**
**Gender**	0.256 (25.6%)	-0.258 (-22.7%)	-0.181 (16.5%)	-	-	-
**Age**	0.037 (3.7%)	0.045 (4.6%)	0.024 (2.4%)	-0.108 (-10.2%)	-	-0.365 (-30.6%)
**Living alone**	0.162 (16.2%)	-	-	-0.532 (-41.3%)	0.133 (14.3%)	-
**Income**	-0.318 (-31.7%)	-0.297 (-25.7%)	-0.302 (26.1%)	-	-0.348 (-29.4%)	-
**Diabetes**	0.360 (36.0%)	-	0.276 (31.8%)	-1.023 (-64.1%)	0.437 (54.9%)	-
**Myocardial infarction**	0.427 (42.7%)	0.588 (80.1%)	0.338 (40.3%)	-	0.454 (57.7%)	-
**Heart failure**	0.351 (35.1%)	0.322 (37.9%)	-	-	-	-
**Stroke**	0.618 (61.2%)	-	0.459 (58.2%)	-	0.543 (72.2%)	-
**Kidney diseases**	1.407 (140.7%)	1.434 (319.6%)	0.338 (40.3%)	-	0.466 (59.4%)	-
**Hypothyroidism**	0.524 (52.4%)	-	-	-	-	-
**Number of missing teeth**	0.032 (3.2%)	0.041 (4.2%)	-	-	-	-
**Oral dryness**	0.060 (6.0%)	-	-	-	-	-
**Likelihood ratio test**	< 0.001	0.001	< 0.001		< 0.001	
**Sum of squared deviations**	1347	1460	1491		**1339**	

It is important to note that overdispersion could lead to the underestimation of standard errors and cause inappropriate assessment of the significance of individual variables. The parameters estimated from the first equation that had a significant negative impact on the quality of life were: living alone (14.3%), diabetes (54.9%), prior myocardial infarction (57.7%), stroke (72.2%) and kidney diseases (59.4%), while income had a positive impact on the quality of life assessed with the EQ-5D questionnaire (-29.4%). The analysis of parameters from the second equation indicates that the independent variable – age – reduces the probability of the best quality of life (EQ-5D = 0). A change in this parameter by 1 year is accompanied by a reduction in the number of patients with the best quality of life by 30.6%. As a synthetic indicator of the measure of count values fit was calculated based on the model with the actual values, the sum of their squared deviations was calculated for each model. The obtained results revealed that the ZINB model, with the lowest sum of squared deviations (1339), was the best of the four proposed models.

*P* Poisson model, *NB* Negative Binomial model, *ZIP* Zero Inflated Poisson model, *ZINB* Zero Inflated Negative Binomial model, e1 – fist equal (Poisson with log link), e2 – second equal (binomial with logit link).

In the Poisson regression model (Table 6), the following unit changes in the analysed parameters adversely affected the expected change in the quality of life assessed with the OHIP-14 questionnaire: the need for oral treatment (16.9%), the number of decayed teeth (5.0%) and the number of missing teeth (4.9%), as well as the number of comorbidities (10.3%) including asthma (49.8%) and reflux (298.3%).

**Table 6:** Parameters of regression models and the impact of unit changes in individual variables on the expected number of patients (%) for the compared OHIP-14 models

Independent variables	P	NB	ZIP		ZINB	
			**e1: Y > 0**	**e2: Y = 0**	**e1: Y > 0**	**e2: Y = 0**
**Gender**	-	-	0.237 (26.8%)	-	0.271 (31.1%)	-
**Age**	-	-	-0.018 (-1.8%)	-	-0.025 (-2.5%)	-
**Income**	-	-	0.116 (12.3%)	-	-	-
**Number of comorbidities**	0.098 (10.3%)	-	-	-	-	-
**Asthma**	0.404 (49.8%)	-	0.158 (17.2%)	-0.935 (60.7%)	-	-0.988 (-62.8%)
**Gastroesophageal reflux disease**	1.381 (298.3%)	-	0.380 (46.3%)	-0.710 (50.8%)	-	-0.765 (-53.4%)
**Oral treatment need**	0.157 (16.9%)	-	0.185 (20.4%)	-	0.278 (32.0%)	
**Number of decayed teeth**	0.049 (5.0%)		0.036 (3.6%)	-	-	-
**Number of missing teeth**	0.048 (4.9%)	0.041 (4.2%)	0.050 (5.1%)	-	0.053 (5.4%)	-
**Likelihood ratio test**	< 0.001	0.002	< 0.001		< 0.001	
**Sum of squared deviations**	87,879	89,349	87,735		**87,358**	

The variance of OHIP-14 was greater (Var = 186.1) than the mean of the variable (Mean = 8.0), overdispersion could cause underestimation of standard errors and inadequate assessment of the significance of individual variables. In the ZINB model, parameters estimated from the first equation that had a significant negative impact on the quality of life included female gender (31.1%), the need for oral treatment (32.0%) and the number of missing teeth (5.4%). The variable ‘age’ had a positive impact (-2.5%). The analysis of parameters estimated from the second equation revealed that asthma and reflux decrease the probability of OHIP-14 = 0 by 62.8% and 53.4%, respectively. The obtained results revealed that the ZINB model, with the lowest sum of squared deviations (87,358), was the best among the four proposed models.

*P* Poisson model, *NB* Negative Binomial model, *ZIP* Zero Inflated Poisson model, *ZINB* Zero Inflated Negative Binomial model, e1 – fist equation (Poisson with log link), e2 – second equation (binomial with logit link).

In the Poisson regression model (Table 7), the following unit changes in the analysed parameters adversely affected the severity of depression assessed with the PHQ-9 questionnaire: female gender (33.0%) and the number of comorbidities (20.3%). The only variable with a positive impact on the number of patients with depression was income (-26.5%). Also, the variance of PHQ-9 (Var = 16.8) was higher than the mean of that variable (Mean = 3.6).

**Table 7:** Impact of unit changes of individual variables on the expected number of patients (%) in the compared models for PHQ-9

Independent variables	P	NB	ZIP		ZINB	
			**e1: Y > 0**	**e2: Y = 0**	**e1: Y > 0**	**e2: Y = 0**
** Gender**	0.285 33.0%	0.322 38.1%	0.174 19.1%	-	-	-
** living with other people**	-	-	-	-	-	0.501 65.0%
** Income**	-0.305 -26.5%	-0.332 -28.3%	-0.179 -16.4%	0.401 49.4%	-0.225 -20.2%	-
** Infarction**	-	-	0.250 28.4%	-	-	-
** Gastroesophageal reflux disease**	-	-	0.728 107.2%	-	0.797 122.0%	-
** Number of comorbidities**	0.184 20.3%	0.203 22.5%	0.044 4.5%	-0.534 -41.4%	-	-0.656 -48.1%
** Number of missing teeth**	-	-	0.007 0.7%	-	-	-
** Likelihood ratio test**	< 0.001	0.001	< 0.001		< 0.001	
** Sum of squared deviations**	7311	7494	7050		**6916**	

Overdispersion could lead to underestimation of standard errors and inappropriate assessment of the significance of individual variables. In the ZINB model, the parameter estimated from the first equation that had a significant negative impact on the occurrence of depression was reflux (122.0%), and income turned out to have a positive impact on it (-20.2%). The analysis of parameters estimated from the second equation reveals that the number of comorbidities decreases the probability of a complete absence of depression (PHQ-9 = 0) by 48.1% while living with other people increases it by 65.0%. The ZINB model, with the lowest sum of squared deviations (6916), was the best among the four proposed models.

*P* Poisson model, *NB* Negative Binomial model, *ZIP* Zero Inflated Poisson model, *ZINB* Zero Inflated Negative Binomial model, e1 – fist equation (Poisson with log link), e2 – second equation (binomial with logit link).

For the dependent variables (EQ-5D, OHIP-14 and PHQ-9), all four count models are significant as a whole. The high values of test statistics for likelihood ratio tests, and, consequently, low values of probability (*p-value* < 0.001), suggest that the null hypothesis about the nonsignificance of the models as a whole should be rejected. The Vuong test was used for comparing the *zero-inflated* class count models with standard count models. The results (p < 0.001) imply that ZIP models are better than standard Poisson models, and ZINB models are better than the standard negative binomial models. *Zero-inflated* class models, i.e. models with an additional equation modelling the probability of the occurrence of zero-value, yield patient counts that better fit the actual (observed) values. The ZINB model, assuming negative binomial distribution in the count equation was found to be the best model in which only 13 independent variables could have a significant impact on the EQ-5D, OHIP-14 and PHQ-9 (Table 8).

**Table 8:** Parameters of regression models (95% CI) for the ZINB models for the EQ-5D, OHIP-14 and PHQ-9

Independent variables	EQ-5D		OHIP-14		PHQ-9	
	e1: Y > 0	e2: Y = 0	e1: Y > 0	e2: Y = 0	e1: Y > 0	e2: Y = 0
Gender	-	-	0.271 (0.015; 0,527) *P* = 0.038	-	-	-
Age	-	-0.365 (-0.611; 0.119) *P* = 0.004	-0.025 (-0.042; 0.008) *P* = 0.003	-	-	-
Living condition	0.133 (0.001; 0.267) *P* = 0.049	-	-	-	-	0.501 (0.125; 0.877) *P* = 0.009
Income	-0.348 (-0.466; 0.230) *P* < 0.001	-	-	-	-0.225 (-0.349; -0.101) *P* < 0.001	-
Diabetes	0.437 (0.250; 0.624) *P* < 0.001	-	-	-	-	-
Myocardial infarction	0.454 (0.151; 0.757) *P* = 0.003	-	-	-	-	-
Stroke	0.543 (0.094; 0.992) *P* = 0.018	-	-	-	-	-
Asthma	-	-	-	-0.988 (-1.833; -0.093) *P* = 0.030	-	-
Gastroesophageal reflux disease	-	-	-	-0.765 (-1.469; -0.061) *P* = 0.033	0.797 (0.115; 1.479) *P* = 0.014	-
Kidney diseases	0.466 (0.206; 0.726) *P* < 0.001	-	-	-	-	-
Number of comorbidities	-	-	-	-	-	-0.656 (-0.877; -0.435) *P* < 0.001
Number of missing teeth	-	-	0.053 (0,039; 0,067) *P* < 0.001	-	-	-
Oral treatment need	-	-	0.278 (0.104; 0.452) *P* = 0.002	-	-	-

e1—parameters of the Eq. 1 to estimate the number of patients with scores greater than zero, e2—parameters of Eq. 2 to estimate the number of patients with scores equal to zero, the parentheses indicate by how much the dependent variable will change with the unit increase of the independent variable, as far as other independent variables will not change

## Discussion

The current approach to identifying the quality of life considers an individual's subjective feeling of meeting their needs, aspirations and values that are important to them. This means that each person determines their own level of life satisfaction. The global population of elderly people is still increasing. Most of the elderly live in developing countries with lower national income, infrastructure and health capacity than developed countries. There is a multilevel and multisector need to improve healthy ageing that prevents diseases and promotes health. The document concerning the Decade of Healthy Ageing 2020–2030 includes the elderly at the center of the plan. The document is based on the Global strategy and action plan on ageing and health (2016–2030). The global strategy includes multisectoral action for an approach to healthy ageing to encourage both longer and healthier lives [21, 22]. The analyses concerning the QoL evaluation, commonly use several terms, such as living conditions, material well-being, the standard of living and lifestyle. However, economic, statistical, social policy, sociological, medical or philosophical literature lacks both a clear definition of those terms and a precise definition of the relationship between them. Living conditions are most frequently defined as the overall circumstances in which society, households or individuals live. The above-mentioned term is frequently defined using four basic elements: the level of economic well-being that guarantees a certain level of material need satisfaction, the degree of provision of housing and communal infrastructure, the degree of provision of social infrastructure (e.g. health care) and the conditions of the environment in which humans live [1].

In the authors’ own study based on the Poisson regression model, factors that had an estimated impact on HRQoL included SES (living conditions, income) as well as gender and age, also general health (diabetes, prior myocardial infarction, stroke, heart failure, kidney diseases, hypothyroidism), and oral health status (xerostomia, the number of missing teeth). Nonetheless, in the ZINB regression model, which turned out to be the most suitable among all the four proposed models, the variables such as gender, heart failure, hypothyroidism, xerostomia and the number of missing teeth were no longer significant at the level *p* < 0.05. In this analysis, the estimated parameters found to have a significant impact on HRQoL were, first of all, SES factors—living conditions (living alone) and income, as well as general health status. Similarly, a study concerning elderly Chinese people found that participants with less subjective economic hardship reported much better self-rated health [23]. Besides, educational level, occupational level and economic indicators (income and expenditure) tended to associate with physical HRQOL only among elderly Chinese men. The subgroup of male participants with the highest education and occupation reported better HRQOL [23]. The study by Huguet et al. conducted on a group of individuals aged 65 or more found that HRQoL was significantly associated with household income in the elderly American population, prompting a search for factors affecting that relationship, including the roles of access to health care and socioeconomic inequalities [24]. According to Pinquart et al., three aspects of life circumstances – SES, social network and competence – are positively associated with subjective well-being in later life. Income was more strongly correlated with well-being than education [25]. Similarly, the analysis of the English Longitudinal Study of Aging (ELSA) indicated that lower SES was related to the acceleration of age-related impairments independently of the diagnosed health conditions or self-rated health [26]. Subjective social status (SSS) and its association with SES indicators and with health outcomes were also assessed among British civil servants (participants in the Whitehall-II study) and U.S. whites and blacks (participants in the CARDIA study) [27]. It was found that SSS was significantly associated with general health and depression in all groups. On the other hand, occupation was a more important determinant of SSS in Whitehall-II than in CARDIA, and among CARDIA participants education and income were the more significant parameters [27]. An attempt was also made to identify [28] mechanisms behind the association between the markers of socioeconomic status (SES) and health in the British Whitehall II and the French GAZEL study. It was found that cultural differences and context-specific characteristics need to be taken into account as they may have an impact on the social distribution of unhealthy behaviors within a population [28]. The impact of the variety of cultural circumstances on gender differences in the aspects of health was also assessed in various countries—11 Continental European countries (Survey of Health, Ageing and Retirement, SHARE), the English Longitudinal Study of Ageing (ELSA) and the Health and Retirement Study (HRS) for the USA [29]. The findings revealed higher levels of heart disease, which is the major cause of mortality in these countries, in men, whereas women are more prone to disabilities, functioning problems and depressive symptoms [29]. Based on the data obtained in the US Health and Retirement Survey (HRS), the Survey of Health, Ageing and Retirement in Europe (SHARE), and English Longitudinal Study of Ageing (ELSA), it was noted that US adults of all socioeconomic levels report worse health than English or other European adults, which allows concluding that differences in health status depend on the place of residence [30]. The results of the presented study indicate that not only SES factors but also general health status are the parameters that have a significant impact on the quality of life assessed with the EQ-5D questionnaire. A negative impact of six chronic systemic diseases (diabetes, myocardial infarction, heart failure, stroke, kidney diseases, hypothyroidism) on HRQoL is observed. Similarly, a comprehensive overview of published EQ-5D index scores in chronic diseases revealed a substantial reduction in HRQoL associated with chronic diseases [31]. The decrease in the quality of life is mainly due to various complaints and pain and there is a close relationship between health status and the quality of life [32]. However, no significant differences were found neither in the prevalence of pain and symptoms of depression nor in the average levels of quality of life in elderly residents of institutions in three European countries—France, Germany and Poland [32]. In the applied Poisson regression model, oral health status (xerostomia, the number of missing teeth) had a negative impact on HRQoL. Consistently with our results, Park et al. found that the number of remaining teeth was associated with QoL assessed with EQ-5D, and subjects who had more teeth obtained higher QoL scores [33].

In our own analysis based on the Poisson regression model, OHRQoL was negatively affected by parameters related to both oral health status (the need for oral treatment, the number of decayed teeth and the number of missing teeth) and general health – mean number of comorbidities, including asthma and reflux. At the same time, in the ZINB model, the parameters that were found to have a significant negative impact on OHRQoL included the female gender, the need for oral treatment and the number of missing teeth. What is more, asthma and reflux significantly reduced the probability of the best OHRQoL. Our data presented that oral health-related quality of life (OHIP-14) deteriorates with the number of missing teeth. This observation is consistent with a study by Masood et al. [34] who found that people aged 65 and older had impaired oral health-related quality of life in comparison to a reference group with 0–5 missing teeth [34]. It is similar to the findings concerning the Chinese elderly population [35]. An attempt was made to assess the factors affecting OHRQoL. Women with 2 or more teeth with root caries reported worse OHRQoL [35]. In our study of the dependent variable OHIP-14 in all four models, it was found that SES factors, such as income and level of education, were significant at the level *p* < 0.05. At the same time, Flemnig et al. observed that the prevalence of complete tooth loss among older adults was higher among adults with lower education [36]. Brazilian researchers evaluated demographic, socioeconomic and dental clinical predictors of OHRQoL in the elderly. Being older was the predictor of a lower educational level but higher income. A higher income, on the other hand, was linked to a better dental status and thus better OHRQoL [37].

The results of the presented study indicated that female gender is a factor that impacts not only OHRQoL but also the severity of depression symptoms. A higher prevalence of depression in women was also found based on the Survey of Health, Ageing and Retirement in Europe (SHARE) [38]. Women experienced worse physical health, had a lower SES, were less likely to be employed and more likely to have been diagnosed with depression in the past [38]. Moreover, an own study using the Poisson regression model revealed that also the number of comorbidities, and the presence of reflux disease in the ZINB model, had an impact on the prevalence of depression assessed using the PHQ-9 questionnaire. Our data showed that gastroesophageal reflux disease (GERD) is associated with depression measured on the PHQ-9 scale. A similar relationship was described previously [39]. Symptoms of depression and anxiety were assessed with the use of the Hospital Anxiety/Depression Scale and were higher in patients with GERD, particularly those who also reported concerns regarding chest pain. The correlation of anxiety and depression with GERD is not fully elucidated. Stress and emotions as well as psychological factors might influence or even cause gastrointestinal symptoms [39]. The results obtained using all four statistical models suggest that material status was a significant positive parameter with an impact on the level of depression assessed with the use of the PHQ-9 questionnaire. Additionally, in the ZINB model, parameters such as the number of comorbidities reduce the probability of avoiding the occurrence of depression symptoms assessed with the PHQ-9 questionnaire, while living with other people increases this probability. Observations conducted by Noguchi et al. [40] led to the conclusion that social isolation is a risk factor for depressive symptoms in older age. At the same time, in the research of Gale et al. [41], loneliness and social isolation have been linked with premature mortality and functional decline in older people. It was found that high levels of loneliness, but not of social isolation, increased the risk of becoming physically frail [41].

Moreover, Rouxel et al. [42], in an analysis of elderly adults living in England (ELSA), found that there is an association between oral health-related quality of life and loneliness. Oral health-related quality of life was identified as an independent risk factor for loneliness amongst older adults. Maintaining good oral health in older age may be a protective factor against loneliness. The results of own study suggest that living with other people increases the probability of avoiding depression symptoms. For elderly adults, loneliness is a factor that scares and frequently takes away their will to live. Therefore, it undoubtedly affects their perception of QoL [42]. The research that evaluated the life satisfaction and the occurrence of mental disorders in elderly people living in the Voivodeship of Podlaskie in Poland allows the conclusion that those who are single or in worse financial standing may have relatively lower life satisfaction and a higher level of depression [43]. A British study attempted to assess the impact of demographic, psychological, health and social variables on loneliness in older people in terms of two dimensions – social and emotional ones. According to that study, male gender, widowhood, low well-being, low self-esteem, low income, very limited contact with family, very limited contact with friends, low activity, low perceived community integration and receipt of community care were significant predictors of social loneliness. Widowhood, low well-being, low self-esteem, high activity limitation, low income and non-receipt of informal care were significant predictors of emotional loneliness [44]. A broadly defined activity on many levels is the most desirable factor for the elderly; it becomes their motive, gives them strength and a will to live. The results of the study by Czech scientists showed that it is necessary to create opportunities for the development and maintenance of social contacts, the involvement of the elderly in various leisure activities and various programmes or voluntary activities. They also stated that treatment of depression and anxiety is very important in terms of improving the quality of life in elderly adults [45]. Active ageing (AA) is crucial for improving the health of populations across Europe. Multidimensional models that include psychosocial, biomedical and external factors will best help detect areas of deficiency and proficiency [46]. The differences in AA in terms of age, education, and occupation were found depending on the country—Spain, Poland, and Finland. Finland scored consistently the highest in AA followed by Spain and Poland. Younger age was associated with higher AA. Higher education and occupation was associated with higher AA. Being married or cohabiting was associated with better AA compared to being widowed or separated in most definitions. Men scored higher in AA only in Spain, whereas there was no gender association in the other two countries. Being widowed was only associated with lower AA in Poland and not being married was associated with lower AA in Poland and Finland but not Spain [46].

Social policy towards the elderly can be generally defined as a system of activities that will support the meeting of the elderly's specific needs and compensate for the decreasing ability of the elderly to meet their needs independently. In the study [47] among community-dwelling people aged 75 + in Poland, two most prevalent social support networks (SSN) types were identified using the Practitioner Assessment of Network Type—“family-dependent” (35.8%) and “locally integrated” (32.2%). Older people with a locally integrated SSN, in contrast to the family-dependent type, were generally younger, living alone and less likely to be homebound, rated their health as poor, suffer from depression or dementia, and had lower levels of functional disability [47]. The key role is played by the family situation of the elderly. Marital status is strongly correlated with gender: while as many as 83.9% of men aged 65–69 and 42.4% of those aged 90 and more were married, the corresponding percentage of women was 55.9% and 0.7%, respectively. Women's widowhood is associated not only with living alone but also with significantly lower income, hence it is difficult for widowed women to meet their needs independently. Nearly half (49.3%) of all older patients stay in one-generation households. Such households are either one-person or two-person households usually run by married couples that belong to the same generation. This indicates the need to analyse both the ability of such households to meet their needs independently and the chances of getting help from family and informal groups. This is particularly the case in the largest cities, where one-generation households constitute almost two-thirds of households, while the share of these households in rural areas is by 50% smaller. The need for help from others is one of the most significant problems accompanying one-generation households. Studies show that an overwhelming number of people who need support due to their reduced mobility benefit from help provided by their family members. However, efforts should be made to rapidly develop a comprehensive system of care and nursing services provided at the place of residence of a person with reduced mobility [3].

Research on the elderly population is not easy due to the diversity of this group in terms of age, health status, disability level, wealth level, family situation and living conditions. Studies are mainly hindered by problems with communication with participants, especially with the oldest ones, as well as the reluctance of individuals to participate in research. Angelini et al. noted that self-assessments in elderly people with health problems and physical limitations concerning their life satisfaction can be potential sources of scale biases due to the reduction of the level of life satisfaction as well as pessimistic bias in the reporting style of the respondents [48]. Besides, several limitations have to be also taken into consideration in a discussion concerning the results of this study. Firstly, this study was conducted using a self-report questionnaire which could lead to identification bias. That is why the selection of adequate tools is so important. Only a validated and reliable questionnaire should be used. The Oral Health Impact Profile – OHIP Questionnaire, which had not been available in the Polish language before, was validated by us. The obtained data indicated the reliability and validity of the OHIP-14 tool for the assessment of the oral health-related quality of life in the Polish adult population [18]. In our own study, results of the analysis concerning the OHIP-14, EQ-5D and PHQ-9 items indicate very good reliability. Secondly, the use of survey data did not allow explanation of the temporal relationships nor inferences on causality. Thirdly, when analysing the obtained results, it is essential to bear in mind a possible self-selection error of the study participants – those who joined the study were concerned about their dental problems or aware of such problems and looking for help. Moreover, some individuals may have refused to participate in this study due to their dental fear. Dental anxiety is more common in edentulous subjects than in the subjects who still have at least part of their natural dentition [49]. Another interfering factor was related to the exclusion criteria. This study was limited by the exclusion of patients with concurrent systemic diseases in whom periodontal probing leading to transient bacteraemia might have posed a risk to their overall health. Transient bacteraemia may occur as the result of many dental procedures and it is well documented following periodontal probing i.a. in patients with periodontitis [50]. Patients with untreated periodontitis are at greater risk of bacteremia due to periodontal probing than patients with chronic gingivitis. For individuals at risk of infective endocarditis, radiographic assessment prior to periodontal probing would be advisable to identify those with periodontitis so that appropriate antibiotic prophylaxis can be provided [50, 51]. Finally, the exclusion of patients with a mental disorder was probably another interfering factor. Several studies have shown that mental disorders have a significant association with non‐response and attrition [52]. In psychiatric epidemiology, this issue is of crucial importance. High response rates tend to alleviate bias whereas low response rates make study validity questionable [52]. The above-mentioned exclusion criterion involved many elderly patients.

## Conclusions

The study concerning elderly residents of the macroregion PL5 (NUTS 1) in the city of Wroclaw PL514 (NUTS 3) found the impact of the socioeconomic status (SES), general health, oral health on health-related quality of life (HRQoL), Oral Health Related Quality of Life (OHRQoL), and depression in older Polish adults. The independent predictors were as follows:

1. SES factors (living conditions, income), as well as age and general health (diabetes, prior myocardial infarction, stroke, kidney diseases,), can have a negative impact on health-related quality of life ( HRQoL) assessed using EQ-5D.
2. Parameters associated with both oral health status (the need for oral treatment, and the number of missing teeth) and general health (asthma, gastroesophageal reflux disease) as well as gender and age can be considered factors negatively affecting OHRQOL.
3. The number of comorbidities can have an impact on the severity of depression, while material status can be a significant positive parameter affecting the level of depression assessed with the PHQ-9 questionnaire. It should be emphasized that living with other people can be a factor significantly increasing the probability of avoiding the occurrence of depression symptoms.

Research on the quality of life of the elderly at the local level allowed to assess the factors linked to quality of life of older adults. Collection of data on the oral health of the Polish elderly population, as well as the chance to examine half a thousand citizens of one of the biggest cities in Poland, clearly indicates the purposefulness of the conducted research and its contribution to science, especially because similar data regarding health condition and the quality of life of the elderly is barely available. The great advantage of the study was the possibility of reaching out to a significant population of the elderly to promote oral health and advise them on preventive measures and treatment options corresponding to their medical needs.

## Data Availability

The datasets used and analysed during the current study are available from the corresponding author on reasonable request.

## Abbreviations

QoL: Quality of Life
OHRQoL: Oral Health-Related Quality of Life
EQ-5D: Euro-Quality of Life
PHQ-9: Patient Health Questionnaire
OHIP-14: Oral Health Impact Profile 14
DMFT: Decayed Missing Filled teeth index
DT: Number of Decayed Teeth
MT: Number of Missing Teeth
FT: Number of Filled Teeth¶BoP- bleeding on probing
PD: Gingival pocket depth
CAL: Clinical attachment loss
SES: Socioeconomic status
